# Monitoring of mechanical errors and their dosimetric impact throughout the course of non-coplanar continuous volumetric-modulated arc therapy

**DOI:** 10.1186/s13014-018-0972-7

**Published:** 2018-02-14

**Authors:** Hideaki Hirashima, Mitsuhiro Nakamura, Yuki Miyabe, Megumi Uto, Kiyonao Nakamura, Takashi Mizowaki

**Affiliations:** 1 Department of Radiation Oncology and Image-applied Therapy, Graduate School of Medicine, Kyoto University, 54 Kawahara-cho, Shogoin, Sakyo-ku, Kyoto, 606-8507 Japan; 2 Division of Medical Physics, Department of Information Technology and Medical Engineering, Human Health Sciences, Graduate School of Medicine, Kyoto University, 53 Kawahara-cho, Shogoin, Sakyo-ku, Kyoto, 606-8507 Japan

**Keywords:** Volumetric-modulated dynamic WaveArc therapy (VMDWAT), Logfile, Dose reconstruction, Monitoring of mechanical errors and their dosimetric impact

## Abstract

**Background:**

Volumetric-modulated Dynamic WaveArc therapy (VMDWAT) is a non-coplanar continuous volumetric modulated radiation therapy (VMAT) delivery technique. Here, we monitored mechanical errors and their impact on dose distributions in VMDWAT using logfiles throughout the course of treatment.

**Methods:**

Fifteen patients were enrolled (2 skull base tumor patients and 13 prostate cancer patients). VMDWAT plans were created for the enrolled patients. The prescribed dose for the skull base tumor was set as 54 Gy at 1.8 Gy per fraction, and that for the prostate cancer was set as 72 to 78 Gy at 2 Gy per fraction. We acquired logfiles to monitor mechanical errors and their impact on dose distribution in each fraction. The root mean square error (RMSE) in the multi-leaf collimator (MLC), gantry angle, O-ring angle and monitor unit (MU) were calculated using logfiles throughout the course of VMDWAT for each patient. The dosimetric impact of mechanical errors throughout the course of VMDWAT was verified using a logfile-based dose reconstruction method. Dosimetric errors between the reconstructed plans and the original plans were assessed.

**Results:**

A total of 517 datasets, including 55 datasets for the 2 skull base tumor patients and 462 datasets for the 13 prostate cancer patients, were acquired. The RMSE values were less than 0.1 mm, 0.2°, 0.1°, and 0.4 MU for MLC position, gantry angle, O-ring angle, and MU, respectively. For the skull base tumors, the absolute mean dosimetric errors and two standard deviations throughout the course of treatment were less than 1.4% and 1.1%, respectively. For prostate cancer, these absolute values were less than 0.3% and 0.5%, respectively. The largest dosimetric error of 2.5% was observed in a skull base tumor patient. The resultant dosimetric error in the accumulated daily delivered dose distribution, in the patient with the largest error, was up to 1.6% for all dose-volumetric parameters relative to the planned dose distribution.

**Conclusions:**

MLC position, gantry rotation, O-ring rotation and MU were highly accurate and stable throughout the course of treatment. The daily dosimetric errors due to mechanical errors were small. VMDWAT provided high delivery accuracy and stability throughout the course of treatment.

**Trial registration:**

UMIN000023870. Registered: 1 October 2016.

**Electronic supplementary material:**

The online version of this article (10.1186/s13014-018-0972-7) contains supplementary material, which is available to authorized users.

## Background

Non-coplanar trajectories enable substantial dose reductions to organs at risk (OARs) compared with coplanar trajectories [1]. Dynamic WaveArc (DWA) is a non-coplanar continuous delivery technique, which is realized by continuous rotation of the radiation source around the horizontal and vertical axes, while the gantry and O-ring rotate continuously without rotating the couch of the Vero4DRT system (Mitsubishi Heavy Industries, Ltd., Tokyo, Japan; and BrainLAB, Feldkirchen, Germany) [2]. Recently, a DWA delivery technique, with intensity-modulated beams and varying simultaneous multi-leaf collimator (MLC) positions and dose rates, has become clinically available as a volumetric-modulated DWA therapy (VMDWAT) [3–5]. Treatment planning studies have demonstrated that VMDWAT provides highly conformal dose distributions compared with coplanar volumetric modulated radiation therapy (VMAT) [3, 5]. Due to the high complexity of VMDWAT delivery, many studies have investigated geometric and dosimetric quality assurance (QA) processes to verify the safety and reliability of VMDWAT clinical applications [6–8]. VMDWAT has been clinically applied at Universitair Ziekenhuis Brussels [4] since 2016, and at Kyoto University Hospital since 2017.

In general, patient-specific dosimetric QA is performed once before starting the course of treatment. Even if the dosimetric QA testing results meet institutional criteria, there is no guarantee that treatment machines will continue to perform correctly throughout the course of treatment. Several studies have reported that daily machine errors were small throughout the course of VMAT treatment [9, 10]; however, it is generally difficult to predict dosimetric impacts on patient anatomy due to mechanical errors. Thus far, no studies have reported monitoring of the dosimetric impact of mechanical errors throughout the course of treatment.

Recently, a dose reconstruction method based on an electronic portal image device (EPID) or logfiles was used to verify the daily dosimetric impact due to mechanical errors [8, 11]. EPID-based dose reconstruction allows the reconstruction of the dose distribution within the patient using the actual fluence that passed through the patient. However, Olaciregui-Ruiz et al. reported that disagreement between the planned and reconstructed dose distributions was observed due to the model error in the back-projection algorithm related to the patient’s anatomy [11]. Logfile-based dose reconstruction also enables evaluation of the dose distribution in the patient’s anatomy. However, logfile-based methods without independent confirmation of the log integrity should be used with caution [12]. Thus far, we independently evaluated the MLC position in the logfile, compared it with the measurement, and confirmed that the detection accuracy was comparable to the measurement for VMDWAT [13]. Here, we continuously monitored mechanical errors and their impact on the dose distribution during VMDWAT using logfiles obtained during treatment.

## Methods

### Patients

Fifteen consecutive patients, including 2 patients who were diagnosed with skull base tumors and 13 patients who were diagnosed with prostate cancer between February and August 2017, were enrolled in this study. They were treated using VMDWAT, performed with Vero4DRT equipment. This study was part of a clinical study to evaluate the feasibility and dose delivery accuracy of VMDWAT for skull base tumors and prostate cancer. Additionally, this study was performed in accordance with the Declaration of Helsinki and was approved by our institutional review board (approval number C1236).

### CT simulation and contouring

A computed tomography (CT) simulation was performed, with patients immobilized by a uni-frame mask (MED-TEC, Orange City, IA, USA) and frameless mask (BrainLAB) for skull base tumors, and by a BodyFix instrument (Medical Intelligence, Schwabmünchen, Germany) for prostate cancer. The patients were examined using two clinical CT scanners (SOMATOM Definition Flash; Siemens Healthcare, Forchheim, Germany; and LightSpeed RT, GE Healthcare, Little Chalfont, UK). Slice thickness was 1.0-mm for skull base tumors and 2- or 2.5-mm for prostate cancer.

For the skull base tumors, the clinical tumor volume (CTV) was defined by a radiation oncologist in our institute to include any suspicious residual tumor. The planning target volume (PTV) was obtained by adding an isotropic margin of 2 mm to the CTV. Eyes, lens, chiasm, brainstem, and both optic nerves were delineated as OARs.

For prostate cancer, the CTV was determined as follows: (1) union of the prostate with the base of the seminal vesicles (SVs) for 10 patients with low- or intermediate-risk prostate cancer; and (2) union of the prostate with the proximal $\frac{2}{3}$ of the SVs for 3 patients with high-risk prostate cancer, according to the D’Amico risk classification [14]. The margin of the CTV to the PTV was defined as follows: (1) 8-mm margins isotropically, except for a 5-mm margin posteriorly (in the rectal direction) and a 6-mm margin laterally and anteriorly, for the 10 patients with low- or intermediate-risk prostate cancer; and (2) 8-mm margins isotropically, except for a 6-mm margin posteriorly, for the 3 patients with high-risk prostate cancer. The rectal and bladder wall were contoured as OARs. The rectal wall was contoured as a 4-mm-thick structure inward from the slice, 10 mm below the prostate apex to 10 mm above the tips of the SVs or prostate base. The bladder wall was contoured as a 4-mm-thick structure inward from the bladder.

### Treatment planning

**Fig. 1:**
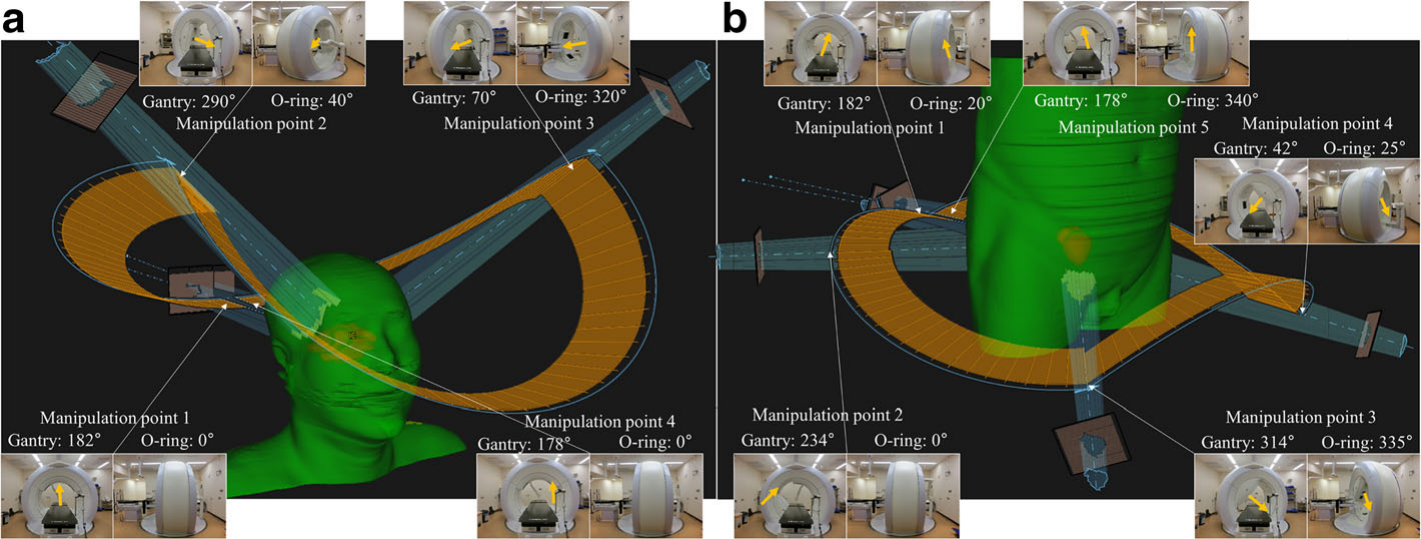
Trajectory of VMDWAT for (**a**) skull base tumor and (**b**) prostate cancer. Orange arrows show the beam direction from gantry to patient. VMDWAT = volumetric-modulated Dynamic WaveArc therapy

VMDWAT plans were created for the enrolled patients using the RayStation (ver. 4.7; RaySearch Laboratories, Stockholm, Sweden) treatment planning system (TPS). There are two steps in VMDWAT planning: (1) selection of the VMDWAT trajectory from among templates on the RayStation TPS: this process partly contributes to the optimization of the dose distribution by selecting the preferred trajectory, which can improve the dose distribution; and (2) intensity modulation and calculation of the dose distribution. VMDWAT planning was done in consideration of the following mechanical specifications: dose rate [150–400 monitor unit (MU)/min)], gantry rotation speed (0.1–6.0°/s), O-ring rotation speed (0.1–2.5°/s), and dynamic MLC leaf speed (1.0–4.0 cm/s). In this study, we selected two non-coplanar trajectories (clockwise gantry direction) with a single arc to meet the institutional dose-volume constraints for the target and OARs (Table 1). The trajectory for the skull base tumors was defined by four manipulation points, where the direction of the gantry and O-ring rotation were switched. Likewise, a trajectory consisting of five manipulation points was selected for prostate cancer. The single arc had 90 control points (CPs), at a minimum of every 4° of gantry angle from 182° to 178° in the clockwise direction. The VMDWAT trajectories for the skull base tumors and prostate cancer are shown in Fig. 1. After optimization, dose distributions were calculated using a collapsed cone with heterogeneity correction carried out with a RayStation TPS. The dose grid size was 1 mm for skull base tumors and 2.5-mm for prostate cancer. The prescribed dose for skull base tumors was set as 54 Gy, at 1.8 Gy per fraction, and that for prostate cancer was set as 72 to 78 Gy at 2 Gy per fraction.

**Table 1:** Summary of dose prescription and dose-volume constraints in VMDWAT planning

Treatment site	Dose-volume constraints		
	PTV	OARs	
Skull base tumor	D_99%_ = 90%	Eye	D_max_ < 45 Gy
	D_max_ < 107%	Lens	D_max_ < 10 Gy
		Chiasm	D_max_ < 55 Gy
		Brainstem	D_max_ < 59 Gy
		Optic nerve	D_max_ < 55 Gy
Prostate cancer	D_mean_ > 99%	Bladder wall	V_40 Gy_ < 65%
	D_95%_ > 90%		V_70 Gy_ < 35%
	V_90%_ > 95%	Rectal wall	V_40 Gy_ < 65%
	D_max_ < 110%		V_60 Gy_ < 35%
			V_70 Gy_ < 25%
			V_78 Gy_ < 1%

*Abbreviations*: *VMDWAT* volumetric-modulated Dynamic WaveArc therapy, *PTV* planning target volume, *OARs* organs at risk, *D*_*mean*_ mean dose, *D*_*max*_ maximum dose *D*_*xx%*_ dose covering xx% volume, *V*_*yy Gy*_ volume receiving yy Gy

### Data analysis

#### Mechanical accuracy throughout the course of VMDWAT

We acquired two logfiles, the MLC logfile and the control logfile, and monitored the machine errors in each fraction. Planned and measured values of MLC position, gantry angle, O-ring angle and MU were recorded every 50 ms in the MLC and control logfiles. To evaluate the geometric accuracy throughout the course of VMDWAT, the mean error and root mean square error (RMSE) were calculated for MLC position, gantry angle, O-ring angle and MU throughout the course of treatment for each patient. The precision values of the logfiles on Vero4DRT were 0.01 mm, 0.1°, 0.1° and 0.1 MU for the MLC position, gantry angle, O-ring angle and MU, respectively.

#### Dosimetric impact due to mechanical errors throughout the course of VMDWAT

Dosimetric impact due to mechanical errors throughout the course of VMDWAT was verified using a logfile-based dose reconstruction method [8]. In-house software searched for the gantry angle, among 90 CPs, corresponding to the planned position, and replaced the planned values of O-ring angle, MLC position and MU in the Digital Imaging and Communications in Medicine–Radiotherapy (DICOM-RT) plan file with the corresponding measured values in the logfiles. Reconstructed DICOM-RT plan files were imported into the RayStation TPS, and dose distributions were recalculated using the planning CT image [8].

The following dose-volumetric parameters were recorded: the dose covering 99% volume (D_99%_), D_1%_ and the mean dose (D_mean_) of the CTV; D_2%_ and D_mean_ of the chiasm and optic nerve; the volume that received greater than 70 Gy (V_70 Gy_); and D_mean_ of the rectal and bladder wall. The dosimetric errors between the reconstructed plans and the original plans were assessed.

For the patient with the largest dosimetric error, the daily delivered plan was summed. The logfile exhibiting the largest dosimetric error was used as a substitute for missing data. For comparison, the whole treatment was simulated only with the logfile exhibiting the largest dosimetric error. Thereafter, we compared the planned dose-volumetric parameters with the accumulated ones.

## Results

### Mechanical errors and their impact on dose distribution throughout the course of VMDWAT

Throughout the course of VMDWAT, a total of 517 datasets, including 55 datasets used for 2 patients with skull base tumors and 462 datasets used for 13 patients with prostate cancer, were acquired. Five and twenty-nine datasets were not recorded for the patients with skull base tumors and prostate cancer, respectively, due to missing data. The patient characteristics, plan information and treatment time for each patient are summarized in Table 2.

**Table 2:** Summary of disease site, prescription dose, PTV volume and delivery parameters

Patient	Disease site	Prescription dose	PTV volume (cm^3^)
1	Skull base tumor	1.8 Gy × 30 fr.	88.8
2	Skull base tumor	1.8 Gy × 30 fr.	54.0
3	Prostate cancer	2.0 Gy × 39 fr.	87.3
4	Prostate cancer	2.0 Gy × 38 fr.	46.2
5	Prostate cancer	2.0 Gy × 38 fr.	77.8
6	Prostate cancer	2.0 Gy × 38 fr.	76.6
7	Prostate cancer	2.0 Gy × 36 fr.	75.4
8	Prostate cancer	2.0 Gy × 38 fr.	49.1
9	Prostate cancer	2.0 Gy × 36 fr.	47.2
10	Prostate cancer	2.0 Gy × 37 fr.	71.3
11	Prostate cancer	2.0 Gy × 38 fr.	61.2
12	Prostate cancer	2.0 Gy × 38 fr.	53.4
13	Prostate cancer	2.0 Gy × 39 fr.	87.3
14	Prostate cancer	2.0 Gy × 38 fr.	53.5
15	Prostate cancer	2.0 Gy × 38 fr.	64.6

*Abbreviations*: *fr* fractions, *PTV* planning target volume

The frequency with which the maximum value of the MLC position, gantry angle, O-ring angle and MU were greater than 1.0 mm, 0.5°, 0.5° and 2 MU were 0.4%, 0%, 0% and 0.6%, respectively (*N* = 517). The mean error values ± two standard deviations (2 SDs) during treatment were − 0.01 ± 0.07 mm, − 0.1 ± 0.1°, 0.0 ± 0.1°, and 0.1 ± 0.3 MU for the MLC position, gantry angle, O-ring angle, and MU, respectively. The mean errors were comparable to the precision values. Moreover, the RMSE values throughout the course of treatment were less than 0.1 mm, 0.2°, 0.1°, and 0.4 MU for MLC position, gantry angle, O-ring angle, and MU, respectively.

**Fig. 2:**
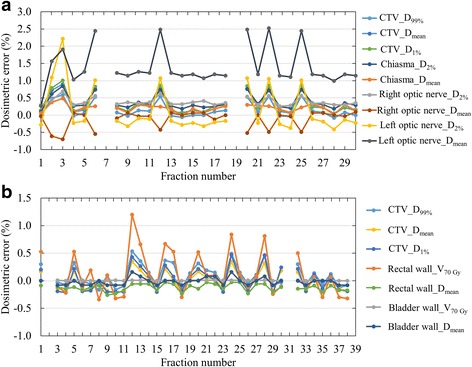
Largest daily dosimetric errors for (**a**) skull base tumor (patient number 1) and (**b**) prostate cancer (patient number 4) throughout the course of VMDWAT. Abbreviations: D_xx%_ = dose covering xx% volume; D_mean_ = mean dose; V_yy Gy_ = volume receiving yy Gy; CTV = clinical target volume

Tables 3 and 4 summarize the mean dosimetric errors ±2 SDs for the skull base tumors and prostate cancer. Figure 2 shows the variations in the dosimetric errors during treatment for skull base tumor and prostate cancer patients exhibiting the largest dosimetric errors. For the skull base tumors, the absolute mean dosimetric errors and 2 SDs throughout the course of treatment were less than 1.4% and 1.1%, respectively. The largest dosimetric error, of 2.5%, throughout the course of treatment was observed in the left optic nerve (Fig. 2a). For prostate cancer, the absolute mean dosimetric errors and 2 SDs throughout the course of treatment were less than 0.3% and 0.5%, respectively. The largest dosimetric error, of 1.2%, throughout the course of treatment was observed in the rectal wall (Fig. 2b).

**Table 3:** Summary of dosimetric errors in the CTV and OARs for skull base tumor throughout the course of VMDWAT. Values are shown in means ± two standard deviations for the signed difference and those for the absolute difference in parentheses

Patient	CTV			Chiasm		Right optic nerve		Left optic nerve	
	D_99%_ (%)	D_mean_ (%)	D_1%_ (%)	D_2%_ (%)	D_mean_ (%)	D_2%_ (%)	D_mean_ (%)	D_2%_ (%)	D_mean_ (%)
1	0.2 ± 0.5 [0.2 ± 0.4]	0.3 ± 0.6 [0.3 ± 0.6]	0.3 ± 0.6 [0.3 ± 0.6]	0.2 ± 0.2 [0.2 ± 0.2]	0.4 ± 0.4 [0.4 ± 0.4]	0.4 ± 0.2 [0.4 ± 0.3]	−0.1 ± 0.5 [0.2 ± 0.4]	0.1 ± 1.3 [0.5 ± 1.0]	1.4 ± 1.1 [1.4 ± 1.1]
2	0.1 ± 0.4 [0.1 ± 0.4]	0.0 ± 0.5 [0.2 ± 0.3]	0.1 ± 0.7 [0.3 ± 0.5]	0.1 ± 0.6 [0.2 ± 0.5]	0.1 ± 0.5 [0.2 ± 0.4]	0.2 ± 0.4 [0.2 ± 0.4]	−0.4 ± 0.3 [0.4 ± 0.3]	0.1 ± 0.4 [0.2 ± 0.3]	0.0 ± 0.3 [0.1 ± 0.2]

*Abbreviations*: *CTV* clinical target volume, *OARs* organs at risk, *VMDWAT* volumetric-modulated Dynamic WaveArc therapy, *D*_*mean*_ mean dose, *D*_*xx%*_ dose covering xx% volume

**Table 4:** Summary of dosimetric errors in the CTV and OARs for prostate cancer throughout the course of VMDWAT. Values are shown as means ± two standard deviations for the signed difference and those for the absolute difference in parentheses

Patient	CTV			Rectal wall		Bladder wall	
	D_99%_ (%)	D_mean_ (%)	D_1%_ (%)	V_70Gy_ (%)	D_mean_ (%)	V_70Gy_ (%)	D_mean_ (%)
3	0.1 ± 0.2 [0.1 ± 0.2]	0.2 ± 0.3 [0.2 ± 0.3]	0.2 ± 0.5 [0.3 ± 0.5]	− 0.3 ± 0.3 [0.3 ± 0.3]	0.0 ± 0.1 [0.0 ± 0.1]	0.2 ± 0.1 [0.1 ± 0.1]	0.0 ± 0.2 [0.1 ± 0.1]
4	0.1 ± 0.4 [0.2 ± 0.3]	0.0 ± 0.3 [0.1 ± 0.2]	0.0 ± 0.4 [0.2 ± 0.2]	0.1 ± 0.8 [0.3 ± 0.5]	−0.1 ± 0.1 [0.1 ± 0.1]	0.0 ± 0.0 [0.0 ± 0.0]	0.0 ± 0.1 [0.1 ± 0.1]
5	−0.2 ± 0.1 [0.2 ± 0.2]	−0.1 ± 0.1 [0.1 ± 0.1]	0.0 ± 0.2 [0.1 ± 0.1]	0.0 ± 0.1 [0.0 ± 0.1]	−0.3 ± 0.2 [0.3 ± 0.2]	0.1 ± 0.1 [0.1 ± 0.1]	0.1 ± 0.1 [0.1 ± 0.1]
6	−0.2 ± 0.1 [0.2 ± 0.2]	−0.1 ± 0.2 [0.1 ± 0.1]	− 0.1 ± 0.3 [0.2 ± 0.2]	−0.3 ± 0.2 [0.3 ± 0.3]	0.0 ± 0.1 [0.1 ± 0.1]	0.0 ± 0.1 [0.0 ± 0.1]	0.0 ± 0.1 [0.1 ± 0.1]
7	0.1 ± 0.4 [0.2 ± 0.3]	0.0 ± 0.2 [0.1 ± 0.2]	0.0 ± 0.3 [0.1 ± 0.2]	0.0 ± 0.0 [0.0 ± 0.0]	0.1 ± 0.2 [0.1 ± 0.1]	0.0 ± 0.1 [0.0 ± 0.1]	0.0 ± 0.1 [0.0 ± 0.1]
8	0.0 ± 0.2 [0.1 ± 0.1]	−0.1 ± 0.2 [0.1 ± 0.1]	−0.1 ± 0.3 [0.2 ± 0.1]	− 0.2 ± 0.2 [0.2 ± 0.2]	−0.1 ± 0.1 [0.1 ± 0.1]	0.0 ± 0.0 [0.0 ± 0.0]	0.0 ± 0.1 [0.0 ± 0.0]
9	−0.1 ± 0.4 [0.2 ± 0.3]	−0.1 ± 0.3 [0.1 ± 0.2]	0.0 ± 0.4 [0.2 ± 0.2]	0.0 ± 0.0 [0.0 ± 0.0]	0.0 ± 0.3 [0.1 ± 0.1]	−0.2 ± 0.1 [0.2 ± 0.2]	−0.2 ± 0.1 [0.2 ± 0.1]
10	0.1 ± 0.2 [0.1 ± 0.2]	0.0 ± 0.1 [0.1 ± 0.1]	0.0 ± 0.1 [0.1 ± 0.1]	0.3 ± 0.2 [0.3 ± 0.2]	0.1 ± 0.2 [0.2 ± 0.1]	0.0 ± 0.0 [0.0 ± 0.0]	0.0 ± 0.1 [0.0 ± 0.1]
11	−0.1 ± 0.2 [0.1 ± 0.1]	−0.1 ± 0.2 [0.1 ± 0.1]	0.0 ± 0.2 [0.1 ± 0.1]	0.1 ± 0.3 [0.1 ± 0.2]	0.0 ± 0.1 [0.0 ± 0.1]	0.1 ± 0.1 [0.0 ± 0.1]	0.0 ± 0.1 [0.0 ± 0.1]
12	−0.1 ± 0.1 [0.1 ± 0.1]	−0.1 ± 0.2 [0.1 ± 0.1]	− 0.1 ± 0.2 [0.1 ± 0.1]	−0.1 ± 0.1 [0.1 ± 0.1]	− 0.3 ± 0.1 [0.3 ± 0.1]	0.0 ± 0.1 [0.0 ± 0.1]	0.1 ± 0.2 [0.1 ± 0.2]
13	−0.1 ± 0.3 [0.1 ± 0.1]	0.0 ± 0.3 [0.1 ± 0.2]	0.1 ± 0.4 [0.2 ± 0.3]	0.2 ± 0.1 [0.2 ± 0.1]	0.0 ± 0.2 [0.1 ± 0.1]	0.0 ± 0.1 [0.0 ± 0.1]	0.1 ± 0.1 [0.1 ± 0.1]
14	0.2 ± 0.3 [0.2 ± 0.3]	0.0 ± 0.3 [0.1 ± 0.2]	−0.1 ± 0.3 [0.1 ± 0.2]	0.2 ± 0.3 [0.2 ± 0.3]	0.0 ± 0.2 [0.1 ± 0.1]	0.0 ± 0.1 [0.0 ± 0.1]	0.0 ± 0.1 [0.1 ± 0.1]
15	0.1 ± 0.2 [0.1 ± 0.2]	0.2 ± 0.2 [0.2 ± 0.2]	0.2 ± 0.2 [0.2 ± 0.2]	0.2 ± 0.2 [0.2 ± 0.2]	−0.1 ± 0.1 [0.1 ± 0.1]	0.0 ± 0.0 [0.0 ± 0.0]	0.0 ± 0.1 [0.0 ± 0.1]

*Abbreviations*: *CTV* clinical target volume, *OARs* organs at risk, *VMDWAT* volumetric-modulated Dynamic WaveArc therapy, *D*_*mean*_ mean dose, *D*_*xx%*_ dose covering xx% volume, *V*_*yy Gy*_ volume receiving yy Gy

**Fig. 3:**
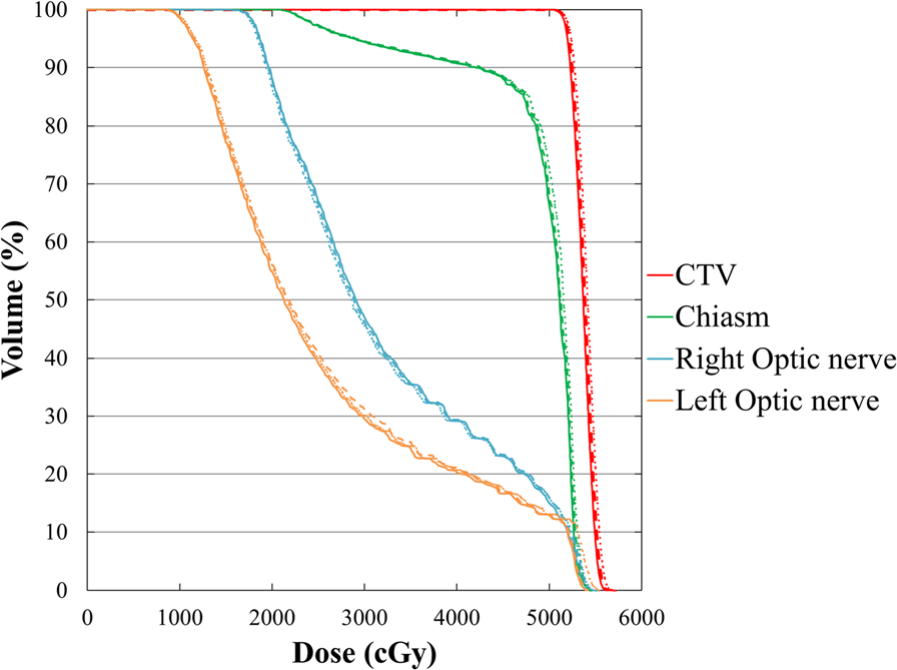
Accumulated DVHs for the skull base tumor patient with the largest dosimetric error (patient 1) are shown. The solid, dashed and dotted lines are the DVHs for the planned, accumulated and largest error dose distributions, respectively. Abbreviation: DVHs = dose volume histograms; CTV = clinical target volume

The dose volume histograms of the accumulated dose distribution for patient 1 with the largest dosimetric error are shown in Fig. 3. The resultant dosimetric errors in the accumulated daily delivered plan and simulation of the whole treatment only with the largest dosimetric error were up to 1.6% and 2.2%, respectively, for all dose-volumetric parameters relative to the planned dose distribution.

## Discussion

An important finding of this study was that mechanical errors throughout the course of VMDWAT were stable for all patients, regardless of MLC movement or non-coplanar trajectory. According to the logfile-based dose reconstruction, the maximum values of the absolute mean dosimetric errors were 1.4% and 0.3% for all dose-volumetric parameters, for skull base tumor and prostate cancer patients, respectively. Monitoring of the dosimetric impact represents a milestone in therapy delivery accuracy.

Scaggion et al. reported that the mean mechanical errors in clinical VMAT plans were less than 1 mm, 0.5°, and 0.1 MU for MLC position, gantry angle, and MU, respectively, during RapidArc treatments delivered with a 6 MV UNIQUE linac (Varian Medical Systems, Palo Alto, CA, USA) over 2 years [9]. José Olasolo-Alonso et al. also reported that the average RMSE values of the MLC were 0.3 mm for Clinac linac (Varian Medical Systems) in intensity-modulated radiation therapy (IMRT) treatments, and 0.04 mm for TrueBeam linacs (Varian Medical Systems) in VMAT treatments, according to 3000 logfiles obtained from four institutions [10]. The mechanical accuracy results observed in this study for VMDWAT, which is a more complex delivery technique compared with VMAT, were similar to those results*.*

Several studies reported that MLC position errors have the most impact on PTV doses, and that there is a strong correlation between the systematic MLC error and dosimetric error [15, 16]. However, we previously reported that dosimetric errors in D_99%_ of the PTV were less than 1%, even though some mechanical errors were detected at the time of QA for VMDWAT; we concluded that mechanical errors had a small impact on dosimetric errors [8]. In this study, the RMSEs were also small throughout the course of treatment. In addition, mechanical errors greater than 1 mm and 2 MU for MLC position and MU, respectively, occurred with a frequency below 1%; such deviations have negligible effects on dose-volumetric parameters throughout the course of treatment. In the optic nerve, a maximum dosimetric error of 2.5% was observed (Fig. 2a). Since a part of the left optic nerve was included in the PTV, the dosimetric impact here would be larger than in other structures. In addition, the dosimetric error for small-volume structures tends to be affected by machine errors [8, 17], thus, this difference in dosimetric error reflected the difference in structure. Even though some daily dosimetric errors exceeded 2%, the resultant dosimetric errors in the accumulated dose distributions were less than 1.6% for all dose-volumetric parameters (Fig. 3). Therefore, the dosimetric impact of mechanical errors throughout the course of treatment was negligible in clinical practice.

There were two limitations to this study. The first was that the reconstructed dose distributions were calculated using planning CT images. In this study, setup errors for skull base tumors were corrected based on bony anatomy using the ExacTrac system (BrainLAB). Since the human head is generally considered a rigid body, the accumulated dose distribution would be almost identical to the planned one, because of small daily dosimetric errors. In contrast, it is well known that the shapes of the prostate (and its surrounding organs) varies among fractions [18, 19]. Deformable image registration is required to reflect such daily stretching and rotation of organs within the daily dose distribution; however, in our institution, cone-beam CT images with a limited field-of-view were acquired daily. Deformation is not guaranteed for limited fields-of-view [20]; therefore, deformation was not considered in this study. The second limitation was that there were only two enrolled disease sites in the present study, and prostate cancer patients accounted for most of them (87%). We performed the planning study to reveal the advantages of VMDWAT for several disease sites before the clinical study. Of those sites, we found that VMDWAT in the current version was suitable for patients with skull base tumors and prostate cancer due to an unmodified trajectory. Thus, the present clinical study included these two disease sites. A new version of RayStation will allow trajectory modification for VMDWAT. Thus, we will clinically apply trajectory-modified VMDWAT to several disease sites other than skull base tumors and prostate cancer and assess treatment plan quality.

## Conclusion

More than 500 VMDWAT sessions were delivered using a 6 MV Vero4DRT, and logfiles obtained at each fraction for 15 patients were analyzed. MLC motion, gantry rotation, O-ring rotation, and dose delivery were highly accurate and stable throughout the course of treatment. Furthermore, dosimetric errors caused by mechanical errors occurred with a frequency below 1.0% in the CTV for skull base tumors and prostate cancer. The largest dosimetric error was observed in an OAR; however, the resultant dosimetric error in accumulated daily delivered dose distribution, in the patient with the largest error, was up to 1.6% for all dose-volumetric parameters compared with the planned dose distribution. The present study demonstrated that VMDWAT with the template trajectories performed using a TPS and Vero4DRT equipment provides high delivery accuracy and stability throughout the course of treatment.

## Additional file

Additional file 1: Dataset supporting our findings. (XLSX 108 kb)

## Abbreviations

CPs: control points
CT: computed tomography
CTV: clinical tumor volume
DICOM-RT: Digital Imaging and Communications in Medicine–Radiotherapy
D_mean_: mean dose
DWA: Dynamic WaveArc
D_XX%_: dose covering XX% volume
IMRT: intensity-modulated radiation therapy
MLC: multi-leaf collimator
MU: monitor unit
OARs: organs at risk
PTV: planning target volume
QA: quality assurance
RMSE: root mean square error
SD: standard deviation
SVs: seminal vesicles
TPS: treatment planning system
VMAT: volumetric modulated radiation therapy
VMDWAT: volumetric-modulated DWA therapy
V_XX Gy_: volume that received greater than XX Gy
